# The Instrument Design of the DLR Earth Sensing Imaging Spectrometer (DESIS)

**DOI:** 10.3390/s19071622

**Published:** 2019-04-04

**Authors:** David Krutz, Rupert Müller, Uwe Knodt, Burghardt Günther, Ingo Walter, Ilse Sebastian, Thomas Säuberlich, Ralf Reulke, Emiliano Carmona, Andreas Eckardt, Holger Venus, Christian Fischer, Bernd Zender, Simone Arloth, Matthias Lieder, Michael Neidhardt, Ute Grote, Friedrich Schrandt, Samuele Gelmi, Andreas Wojtkowiak

**Affiliations:** 1 Institute of Optical Sensor Systems, DLR, Rutherfordstraße 2, 12489 Berlin, Germany; burghardt.guenther@dlr.de (B.G.); ingo.walter@dlr.de (I.W.); ilse.sebastian@dlr.de (I.S.); thomas.saeuberlich@dlr.de (T.S.); ralf.reulke@dlr.de (R.R.); andreas.eckardt@dlr.de (A.E.); holger.venus@dlr.de (H.V.); c.fischer@dlr.de (C.F.); bernd.zender@dlr.de (B.Z.); simone.arloth@dlr.de (S.A.); matthias.lieder@dlr.de (M.L.); michael.neidhardt@dlr.de (M.N.); ute.grote@dlr.de (U.G.); friedrich.schrandt@dlr.de (F.S.); samuele.gelmi@dlr.de (S.G.); andreas.wojtkowiak@dlr.de (A.W.); 2 Remote Sensing Technology Institute, DLR, Oberpfaffenhofen, 82234 Weßling, Germany; rupert.mueller@dlr.de (R.M.); emiliano.carmona@dlr.de (E.C.); 3 Department of Strategic Services, DLR, Linder, Höhe, 51147 Köln, Germany; uwe.knodt@dlr.de

**Keywords:** DESIS, ISS, MUSES, hyperspectral, camera

## Abstract

Whether for identification and characterization of materials or for monitoring of the environment, space-based hyperspectral instruments are very useful. Hyperspectral instruments measure several dozens up to hundreds of spectral bands. These data help to reconstruct the spectral properties like reflectance or emission of Earth surface or the absorption of the atmosphere, and to identify constituents on land, water, and in the atmosphere. There are a lot of possible applications, from vegetation and water quality up to greenhouse gas monitoring. But the actual number of hyperspectral space-based missions or hyperspectral space-based data is limited. This will be changed in the next years by different missions. The German Aerospace Center (DLR) Earth Sensing Imaging Spectrometer (DESIS) is one of the new currently existing space-based hyperspectral instruments, launched in 2018 and ready to reduce the gap of space-born hyperspectral data. The instrument is operating onboard the International Space Station, using the Multi-User System for Earth Sensing (MUSES) platform. The instrument has 235 spectral bands in the wavelength range from visible (400 nm) to near-infrared (1000 nm), which results in a 2.5 nm spectral sampling distance and a ground sampling distance of 30 m from 400 km orbit of the International Space Station. In this article, the design of the instrument will be described.

## 1. Introduction

Hyperspectral remote sensing combines the benefits from remote sensing and spectroscopy. By measurements of several dozens up to hundreds of spectral bands, physical properties like reflectance, emission, or absorption of material can be characterized. These spectral signatures support the identification of elements or the measurement of concentrations. Remote sensing capabilities extend spectroscopy from a local measurement to a global Earth coverage measurement method for the identification of atmospheric, land, and water constitutions [1]. Typical applications of hyperspectral remote sensing are, amongst others, monitoring of the vegetation, forestry and agriculture [2,3], coastal and inland water [4], mining, geology [5], and soils [6]. Since the late 1980s, airborne-based hyperspectral remote sensing instruments have been developed and used in a lot of experiments. Thus, hyperspectral surveys have become a standard for semi-quantitative and quantitative assessments of land surface properties. They are typically working in a spectral range of 400 nm to 2500 nm, covering the visible, near-infrared, and shortwave infrared spectral range. One of the first instruments was the Airborne Visible/Infrared Imaging Spectrometer (AVIRIS), developed by NASA JPL [7]. Since the 2000s, space-based hyperspectral remote sensing instruments have been launched. The first instrument was Hyperion, launched in 2000 as part of the NASA Earth Observing-1 mission [8]. The spectrometer had a ground sampling distance of 30 m, a spectral sampling distance of 10 nm from 400 nm to 2500 nm with 220 bands from a 700 km orbit. Up to now, some, but not too many, additional satellite-based hyperspectral instruments have been launched: Proba-1 in 2001 [9], MERIS on ENVISAT in 2002 [10], HySI in 2018 [11], and PRISMA in 2019 [12]. Additional missions are in planning, like EnMAP [13] (launch planned end of 2020) or SHALOM [14]. With the commissioning of the international space station end of the 1990s, a new platform also suitable for Earth observation was established. The leading instrument in the context of the International Space Station (ISS) was the Hyperspectral Imager for Costal Ocean (HICO) [15], operating from 2009 to 2015. It proved the capability of the ISS for Earth observation. Meanwhile some other instruments are operating onboard the ISS, e.g., ECOSTRESS [16], or are planned to be installed during the next few years, e.g., HISUI [17]. However, the ISS is not optimal for Earth observation. With an inclination of 51.6°, the whole planet cannot be covered. Nevertheless, 90% of the populated surface of the Earth can be observed with an average repetition rate of three to five days. Furthermore, the non-sun synchronous orbit has a negative impact on the illumination conditions. In comparison to sun synchronous orbits, the illumination conditions are different for each orbit. This is very challenging for atmospheric correction algorithms. Another challenge is the complex thermal environment of the instruments onboard the ISS. The variability of the beta angle, the smallest angle between the ISS orbital plane, and the vector to the Sun, in combination with the flexibility of ISS structure elements (turning solar panels and radiators) complicates the thermal design and thermal analysis. However, effort and cost for the development and operation in space of the instruments can be extremely reduced by using the ISS infrastructure to outweigh these disadvantages.

## 2. MUSES Platform

**Figure 1:**
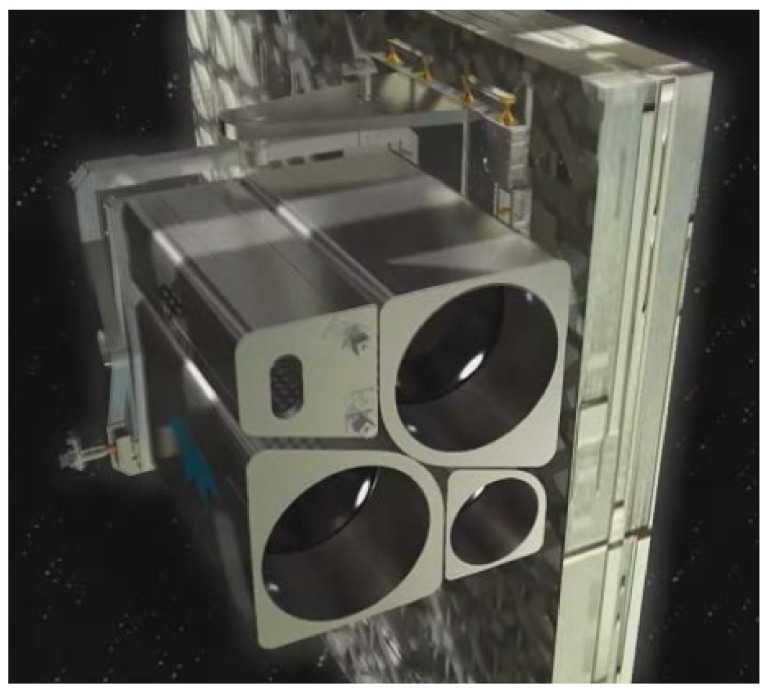
Multi-User System for Earth Sensing (MUSES) platform with the two small and two large slots for payloads. DESIS will be located in one of the large slots.

In 2017, with the integration of the Multi-User System for Earth Sensing (MUSES) platform onboard the ISS, a new milestone for Earth observation from the ISS was reached. The MUSES platform is an Earth-pointing platform for Earth observation instruments. The platform was developed by Teledyne Brown Engineering. Up to four payloads, or instruments, can be integrated on the MUSES platform (see Figure 1). The MUSES system consists of two parts: a platform outside connected to Express Logistics Carrier (ELC-4; the ELC is an unpressurized attached payload platform on the ISS) and a server system inside the ISS for commanding of the platform, massive data storage, and for controlling the image transfer to the ground. The platform can rotate the payloads with an integrated two axis gimbal: ±25° forward/backward view and 45° backboard view and 5° starboard view. This is a big benefit of the MUSES platform. Other instruments onboard the ISS have to integrate the pointing capability inside the instruments. This increases the cost and complexity of the design. The pointing knowledge of the MUSES platform is better than 400 m. This is reached by using an additional star tracker and Miniature Inertial Measurement Unit (MIMU) on the same platform as the payloads. A Gb Ethernet interface is used for the communication between the MUSES platform and the payloads. Groups of Earth observation applications can be created by extending the MUSES platform with new optical instruments and data fusion algorithms.

## 3. DESIS

The first instrument on the MUSES platform is DESIS. It is a hyperspectral instrument, jointly developed by the German Aerospace Center (DLR) and Teledyne Brown Engineering [18]. In addition to the many objectives and applications of hyperspectral missions mentioned above, DESIS is opening up further special fields of application. The off-nadir capability of DESIS with ±15° along the track during a datatake enables investigations of the BRDF (Bi-Directional Reflectance Distribution Function) of objects on Earth and thus provides additional target-specific information. The variable recording times due to the non-polar or non-sun-synchronous orbit allow investigations of solar-induced chlorophyll fluorescence and photosynthesis, which is subject to strong daily fluctuations [1]. And as soon as additional sensors are placed on the MUSES platform, multi-modal observations become possible, which allow sensor fusion approaches employing the same sun-target-sensor geometry, the same atmospheric conditions, and the same object properties. DESIS can also be seen as a precursor instrument for the Environmental Mapping and Analysis Program (EnMAP), as it has the same detector in the visible near-infrared (VNIR) range. The experience gained, especially in the laboratory calibration and commissioning phase of DESIS, will be incorporated into the EnMAP program.

**Figure 2:**
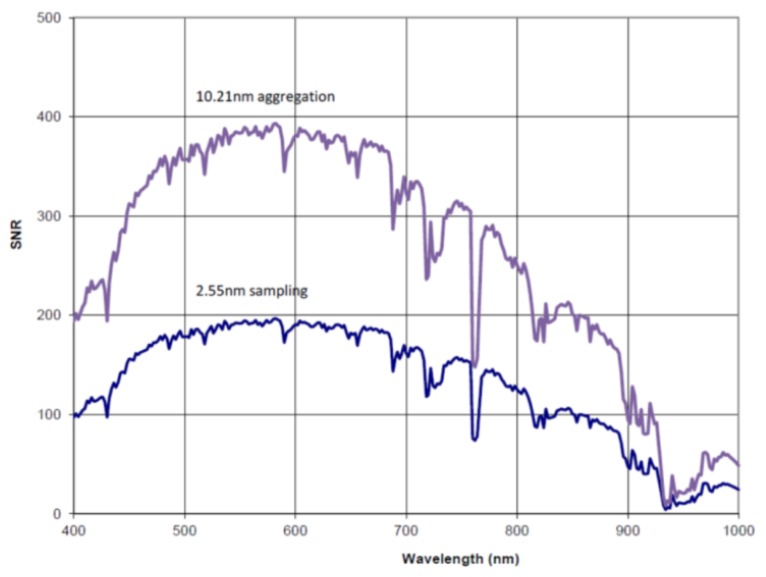
The Signal-to-Noise Ratio (SNR) for spectral sampling without binning (2.55 nm, blue) and binning mode 4 (10.21 nm, purple) at 232 Hz. Simulation based on MODTRAN (MODerate resolution atmospheric TRANsmission) with standard mid-latitude summer atmosphere (Albedo 0.3) and solar zenith angle of 45°.

DESIS system parameters were defined as shown in Table 1. The Signal-to-Noise Ratios (SNR) for unbinned and binning mode are shown in Figure 2. To guarantee the optical performance over the expected mission time of five years, the instrument was equipped with a calibration unit based on LEDs. Two calibration modes are supported: in-orbit spectral calibration, which checks the spectral calibration, and in-orbit radiometric calibration, which monitors the pixel response degradation. In comparison to satellite instruments, DESIS is not able to look at the Sun, Moon, nor deep space for absolute calibrations.

DESIS was launched in June 2018 with SpaceX-15. After the robotic integration of the DESIS instrument to the MUSES platform in August 2018, a commissioning and validation phase was performed. In early 2019, the operational phase started. From specification to delivery, the instrument was developed over a period of just three and a half years.

## 4. DESIS Design

**Figure 3:**
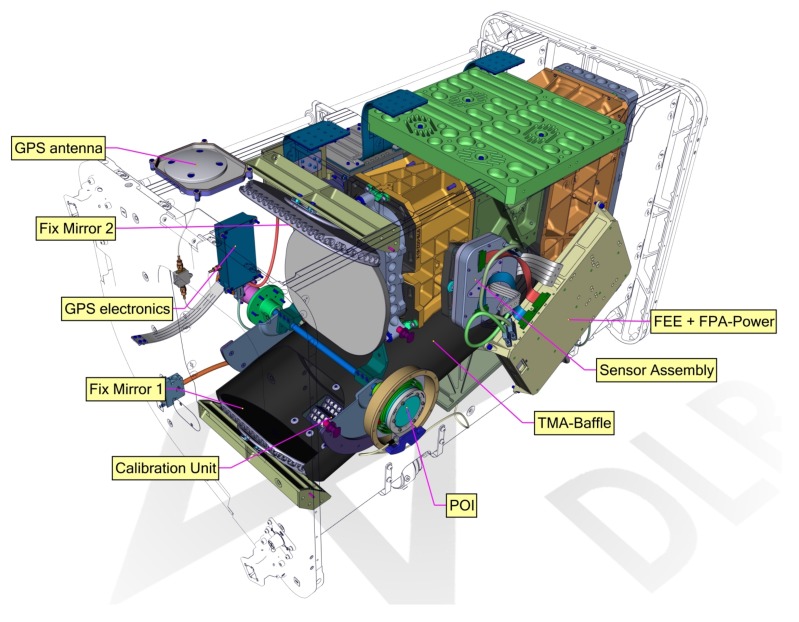
DESIS instrument.

The DESIS instrument design was optimized for optical performance. To increase the radiometric performance, the aperture has the maximum size (i.e., the F-number was minimized), which still fits in the envelopment for MUSES payloads. There are quite a lot of subsystems integrated in the DESIS instrument: spectrometer optics with auxiliary optical components (fix mirrors, baffle), focal plane array (FPA), instrument control unit (ICU) together with the power supply (PCHU), calibration unit, pointing unit (POI), and the container (see Figure 3). The subsystems will be presented in the following sections.

### 4.1. Spectrometer Optics

**Figure 4:**
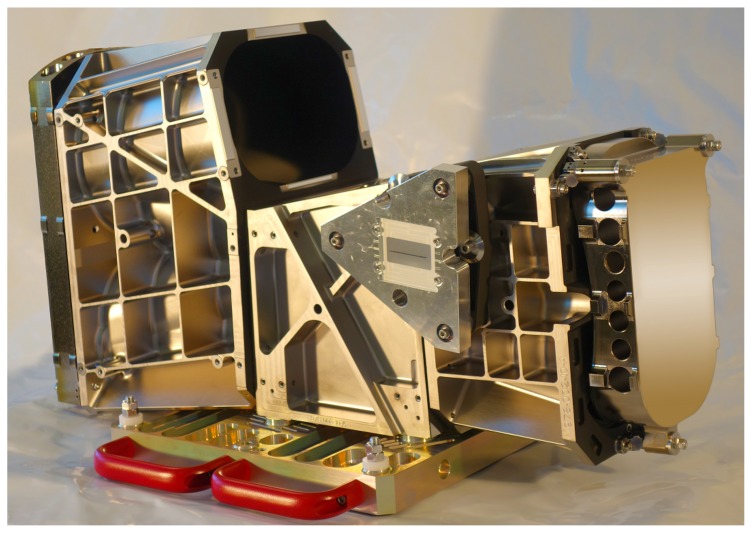
Case of spectrometer optics.

**Figure 5:**
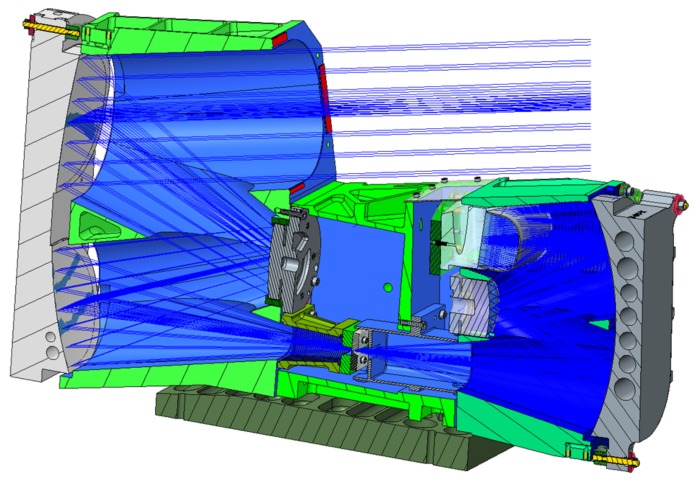
Cut-view of the DESIS opto-mechanical system. The Three-Mirror-Anastigmat (TMA) is on left side, and the spectrometer is on the right [19].

**Figure 6:**
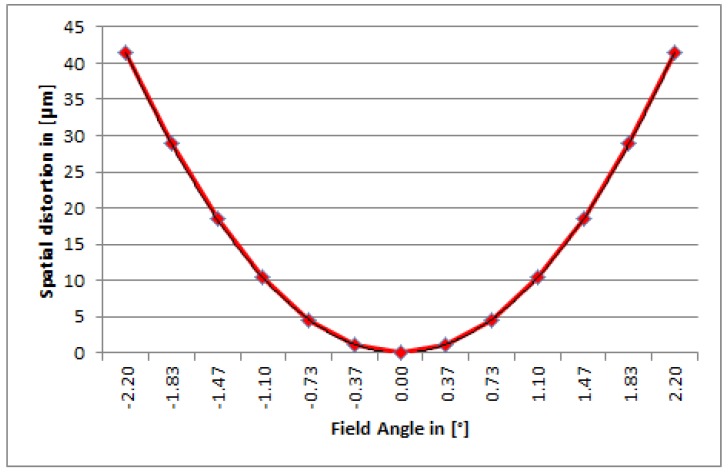
Smile effect @ 700 nm.

**Figure 7:**
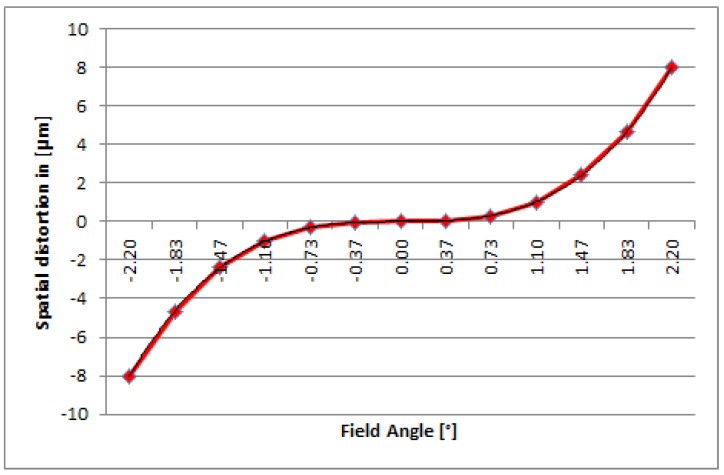
Spectral keystone effect @ 700 nm.

The imaging performance and quality of the DESIS instrument are mainly defined by the optics according to the detector. The DESIS optics, fully integrated within its mechanical structure, is presented in Figure 4 and Figure 5. The optics system relies on metal-based mirrors and is designed as an athermal configuration for a wide temperature ranges onboard the ISS. It consists of a Three-Mirror-Anastigmat (TMA) telescope in front, combined with an Offner-type spectrometer. The optics are designed and manufactured by Fraunhofer IOF in Jena. All three mirrors of the TMA are standard aspheres aligned on a single optical axis. The Offner mirror and TMA mirrors are manufactured by Single-Point Diamant Turning (SPDT), using Subsequent Magneto-Rheological Finishing (MRF), and a post-polishing process. The mirrors M1 and M3 of the TMA [19] are located on a common base substrate structure. They share a common vertex and have been grinded in one work stage that simplified the manufacturing and alignment process of the TMA. The imaging quality of the telescope front optics (TMA) of DESIS is close to the diffraction-limit at the F-number of 2.8. The Offner type spectrometer is made as an excellent re-imaging system, using a convex grating manufactured by SPDT, as well. The grating period is taken with 0.07489 Lines/μm or 13.3 μm. The grating is configured as a binary groove profile. This profile is optimized to improve the first order diffraction efficiency and to suppress the unwanted second order to less than 2%. The achieved diffraction efficiency is measured to be 36% within the first order of the grating and is thus close to the theoretical limit. The Offner mirror itself is using a freeform surface, while the freeform part of the surface is given in terms of Zernicke polynomials. The Offner mirror is symmetrically used twice, by the incoming and outgoing beam, respectively. Due to its freeform shape, a good imaging quality of the spectrometer in combination with a high throughput could be ensured over the whole spectral range. An additional order sorting filter in front of the detector has been integrated to improve the suppression of the remaining second order influence by a factor of 100. Finally, the resulting 2nd order suppression achieves the level of 1:10,000 due to the combination of the grating binary groove shape with an order sorting filter effect. To ensure the stray-light suppression for DESIS optics at high level, a couple of construction parts were implemented into the structure design. The effects of external sources outside the field of view, of unused zero and higher diffraction orders of the grating, and of the internal scattering on the DESIS useful signal are minimized this way. A front baffle-structure coated with Acktar™ Black [20], as well as the detector baffle, was placed in front of the complementary metal oxide semiconductor (CMOS) detector. The slit assembly is especially mechanically designed and is partly coatless and partly coated to fulfill both likewise, stray-light suppression and thermal requirement of heat absorption prevention. The conjugated optical surfaces are individually baffled. DESIS optical analysis shows a low smile effect of 1.7 pixel and a very low keystone effect of 0.3 sub-pixel, each over the whole DESIS field of view and close to identical for all wavelengths. Both, smile and keystone, finally result in special distortion on the focal plane given by position deviations on the focal plane. Figure 6 and Figure 7 show smile and (spectral) keystone as (spatial) distortion versus field angle of DESIS given for the wavelength of 700 nm and derived from the DESIS optical design. The polarization in all directions of the DESIS optical system is required to be smaller than 5%. This value is understood as the maximum of radiometric instrument sensitivity variation dependent on the directionality of Earth reflectance and of transmitted photons from the atmosphere due to scattering effects (Rayleigh scattering from molecules, Mie scattering from aerosols, and Mie scattering from water droplets in clouds). The atmospheric contribution to polarization is, therefore, important and strongly dependent on wavelength due to the large contribution from Rayleigh scattering within the blue range.

To ensure the athermal imaging quality of DESIS, a special aluminum–silicon alloy, together with nickel coating, are used for the structure and for the mirrors, respectively. This allows the full performance range of imaging quality for the temperature range from 15 °C to 25 °C. The mass of the DESIS optics unit is 21 kg.

### 4.2. Focal Plane Assembly

**Figure 8:**
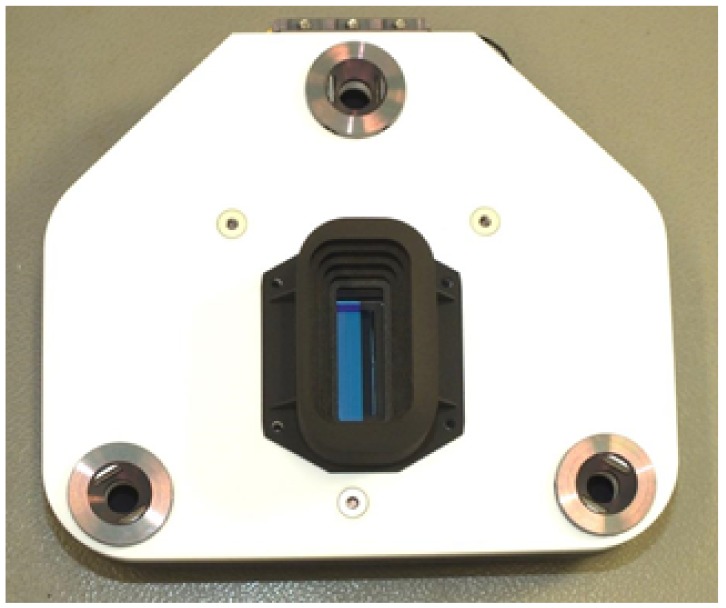
Focal plane assembly of DESIS.

The Focal Plane Assembly (FPA) with the detector is the last element in the optical beam path of the DESIS imaging spectrometer (see Figure 8). It converts detected photons into digital values. Besides the detector as the FPA core element, the proximity electronics for detector operation (BIAS voltage generation), as well as housekeeping monitoring (temperature, voltages, and currents), are allocated. These housekeepings are used for detector temperature stabilization and operation monitoring (latch up detection) in the cosmic radiation exposed environment. To fulfill the optical performance and the mission constraints, a back-illuminated scientific CMOS detector array (CIS2001) from BAE Systems Imaging Solution, Inc., is used. This detector is sensitive from 400 to 1000 nm and has a dimension of 256 × 1056 pixels. Two detector lines are read out in parallel. DESIS uses an array of 235 pixels along track mapping the spectral information and 1024 pixels across track providing the spatial resolution. This number is limited by the detector maximal frame rate. With the ISS given orbit altitude around 400 km and the resulting DESIS Ground Sampling Distance (GSD) of 30 m, the frame rate has to be 232 Hz in order to capture square pixel without gaps or oversampling. This is achieved by limiting the frame to 235 lines (corresponding to spectral bands) and operating in rolling shutter mode. In this mode, each spectral channel is integrated for the same time period, but the line-by-line delayed start of the integration period leads to a shift of the imaged ground sample for each spectral band. The resulting spectral shift is 1/118 pixel size for the second spectral line and 117/118 pixel for the last 235th spectral line. This is the downside of the rolling shutter mode because all spectral bands have to be calculated for the same point on Earth before spectral unmixing can be performed. This remapping of the bands limits the performance of unmixing algorithms. The FPA also supports a global shutter mode (GSM) that avoids the spectral sampling shift. However, the detector is read out twice in GSM (reset image and data image) in order to realize a digital correlated double sampling. This splits the effective GSM frame rate to 117.5 Hz with the consequence of a reduced along track GSD of 60 m. A second drawback of the GSM is an increased sensitivity to low frequency noise due to the two subsequent readouts. The nominal DESIS operation mode is therefore the rolling shutter mode.

### 4.3. Instrument Control and Mass Memory

The interface between the MUSES platform and all subsystems of DESIS is controlled by the Instrument Control Unit (ICU). It consists of a processor board, a network interface board (for the Gb Ethernet to MUSES), and a field-programmable gate array (FPGA) board. Additionally, there is a power supply (PCHU) for the generation of secondary powers of the ICU, power control of all subsystems, and the heater power management. The main function of the FPGA board is the implementation of the mass memory system. The major requirements of the mass memory system of DESIS are: storage capability of 64 GB in NAND flashes, 1 Gb/s write performance of two ChannelLink interfaces, read performance of 70 Mb/s (including Secure File Transfer Protocol (SFTP) data encoding), single event upset (SEU) detection, and correction and wear leveling. The architecture consists of a radiation qualified P2020 processor with Linux operating system (buildroot version) and a Xilinx Virtex-4 FPGA board, connected via Peripheral Component Interconnect (PCI) interface. The FPGA board contains eight 3D Plus NAND flash parts, each consisting of 8 dies of 8 Gb, for a total of 64 GB. The parallel usage of the eight parts in combination with an eight die write-interleave mode guarantees the 1 Gb/s writing performance from 40 MHz parts operating at 33 MHz. On the other side, the parallel and interleave mode complicates the data management. Image frame data is distributed over a lot of pages of all flash dies. To support the reconstruction, one page in each group of 64 pages is dedicated to housekeeping information. It is the software’s responsibility to reorganize the data during reading. The entire file system can be reconstructed after a power cycle by reading the housekeeping information pages. The 64 GB file system takes about 20 s to be initialized. As stored image files are possibly larger than the memory available to the P2020, image data is dynamically reconstructed from the flash and transferred via SFTP. Usage of the P2020/800 MHz processor also helps to fulfill the read performance requirement. The data writing functionality is fully integrated in the FPGA. The processor is only responsible for providing information about the next blocks to write. Cyclic redundancy check (CRC) codes for SEU detection are calculated online and added to the page tail. SEU detection while reading the data is also implemented in the FPGA. However, error correction will be done by the processor. Open-source SFTP server software is used on Linux to reduce the implementation effort. A new file system was developed to close the gap between the operating system file access and the FPGA over PCI. An already existing file system design could not be used because of the direct writing operation of the data without going through the processor. But the file system definition uses concepts of existing journal file systems, like Yaffs (Yet Another Flash File System).

### 4.4. GPS

The DESIS also incorporates a low-cost Phoenix Global Positioning Systems (GPS) receiver, based on the Zarlink Chip Set GP4020. The GPS receiver is working as a time calibration unit. For georeferencing of the images in the order of 1 m (1/3 pixel), an accuracy of the time stamps on each image of 1.5 ms is necessary. The baseline time source is an ISS GPS receiver located on top of the ISS so that the visibility to GPS satellites is excellent. However, the propagation errors (jitter and latency) from the ISS GPS receiver to the instrument are unknown, yet remain very important for the instrument performance. To minimize the risk, the DESIS GPS subsystem was implemented. But with a location of the DESIS GPS receiver below the ISS structure (see Figure 1 and Figure 3) the visibility of GPS satellites is limited. An analysis shows that only in 15% of time more than three GPS satellites are visible and a time locking is possible. However, this short time is sufficient for the calibration of the latency and the measurement of the jitter.

### 4.5. Calibration Unit

**Figure 9:**
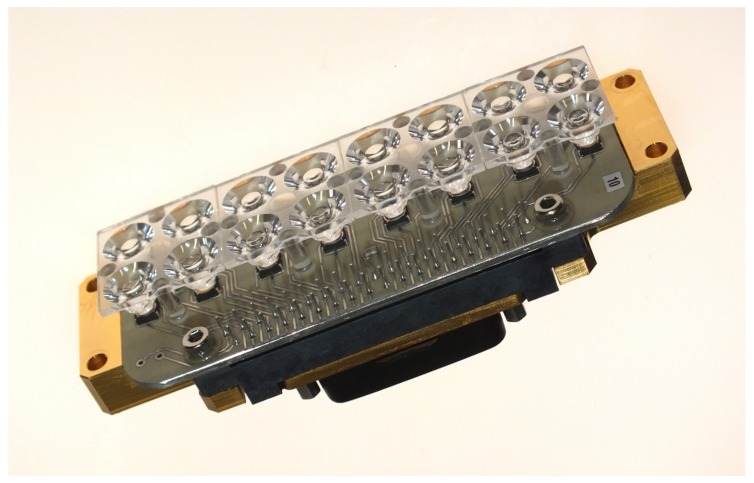
Calibration unit.

**Figure 10:**
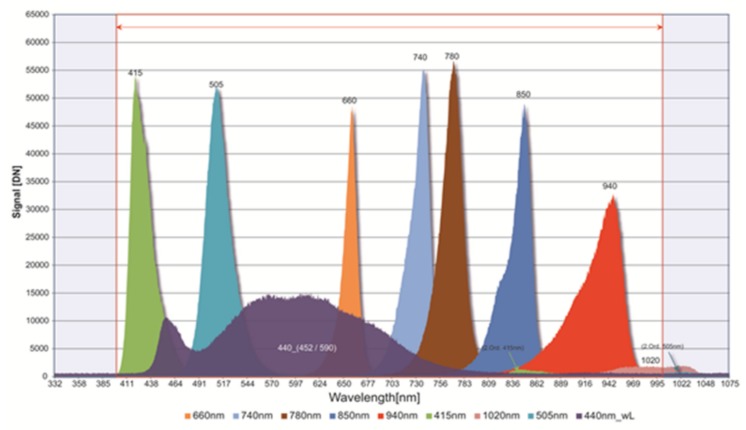
Spectrum of DESIS calibration unit using different LEDs.

**Figure 11:**
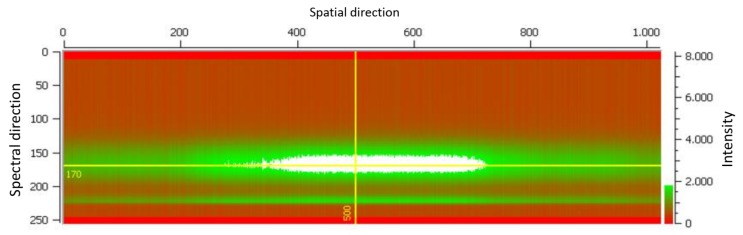
Image on DESIS of white LED calibration.

The inflight spectral and radiometric onboard calibration [21] of the DESIS instrument will be performed by the calibration unit. The line of sight of the whole instrument can be changed by the pointing unit to calibration unit view. The calibration unit consists of two LED bank arrays (see Figure 9). Each bank has nine different types of LED (see Figure 10 for the spectral coverage of the LED types). In front of each LED, Commercial-Off-The-Shelf (COTS) Integrated Micro Optical Systems (IMOS) lenses are used for collimating the light to a cone of ±16°. This cone is not optimal in relation to the instrument field of view of 4.1°, but the usage of a delta-qualified COTS reduced the development time and costs. The LED banks are temperature stable to ±0.5 K because the LED intensity and spectral response are dependent on the temperature. The inflight spectral calibration checks the spectral mapping of the instrument. All nine different LEDs will be switched on (see Figure 11). The measured center of the illumination can be used to check the stability of the center wavelength mapping in the order of 0.5 nm. The inflight radiometric calibration checks the changes on the Pixel Response Non-Uniformity (PRNU). In this mode, a different combination of LED intensities will be used to measure the response of each pixel. The result is used to cross-calibrate the PRNU of the detector.

The LED types used in the calibration unit were tested for Displacement Damage using proton radiation at 30 MeV and for Total Ionization Dose (TID) effects using Cobalt-60 gamma radiation source. The TID effects are negligible below the tested range up to 3.2 kRad(Si). All LEDs are stable in peak and dominant wavelengths as well as in their spectral bandwidth after proton radiation. However, the maximum peak intensity changed as a result of Displacement Damage. For most of the LED types the intensity increased by 1% after $3.2\times 10^{10}\;\frac{proton}{cm^{2}}$, though, for two types, the intensity decreased by 4%. This effect can be used to distinguish between detector and LED degradation and can help to identify the pure detector degradation. Under the same test conditions, the Institute for Micro and Sensor Systems (IMOS) lens showed a reduction of transmission of 0.6%.

### 4.6. Pointing Unit

**Figure 12:**
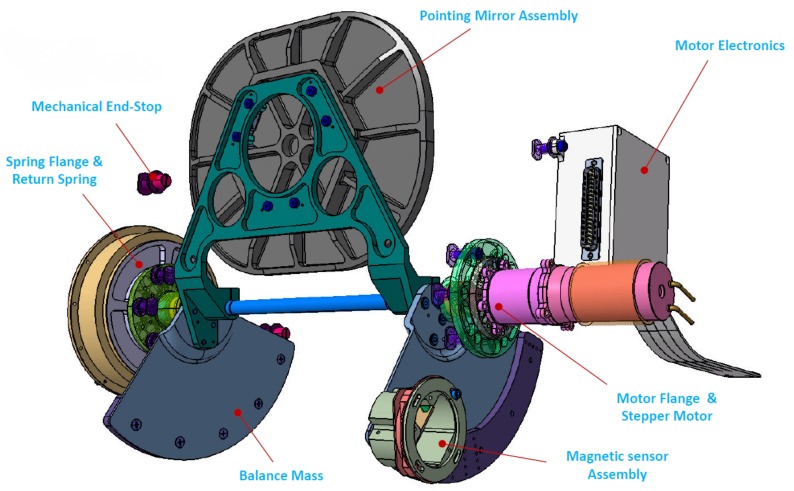
Pointing Unit.

Changing the line of sight of the instrument is the function of the pointing unit (see Figure 12). To achieve this, a plane mirror is moved with the help of a stepper motor, which can vary the line of sight between ±15° at a repeat accuracy of 0.001° ($\frac{1}{4}$ of pixel). With this unit, DESIS has two different operation modes: Earth observation and forward motion compensation. The first mode is used for normal image acquisition and for calibration measurements. In the forward motion compensation mode, the stepper motor turns with a constant speed, which results in a decreasing relative speed of the ground pixels. With this technique the integration time respective to the SNR can be increased. In the Earth observation mode, the stepper motor operates in a half-step mode. This guarantees a good repeatability. In forward motion compensation mode, an 8-fold micro stepping mode will be used. This reduces the smearing effect of the motor steps. The speed is programmable from 0.3${}^{\circ }$/s to 0.75${}^{\circ }$/s with an accuracy of 0.001${}^{\circ }$/s. The high accuracy of the pointing unit is reached by a gear between the stepper motor and the pointing mirror axle with a ratio of 1:288. A counterbalance mass compensates the mirror inertia. To reduce the backlash of the system, a permanent return spring is integrated in the unit. This is also a fallback solution in the case of any electrical failure in which the spring moves the pointing mirror back in the so-called launch position. In the launch position, Earth observation data acquisition can be done without using the pointing unit.

### 4.7. Container and Thermal Design

The container is the structure where the instruments and all components are attached and protected. The outer surface isolates DESIS thermally from other potential instruments on the MUSES platform. Besides this, it provides both mechanical interfaces, to the MUSES platform and the robotic arm of the ISS, while transferring to and installation on MUSES. To reach a high stability of the line of sight, the influence of the thermal environment to DESIS was minimized. The container material has a coefficient of thermal expansion (CTE) compatible to the rest of the DESIS mechanical design. The same approach was also applied to the optics, which follows an athermal design idea with a highly polished, highly planar mechanical interface and with high linear conductive values to each other. A contribution of temperature gradients to the instrument is prevented by a single-point connection between the optics and the container. A third group which influences the line of sight stability is the mechanical stability of the front mirrors (pointing mirror, fixed mirrors). This is achieved by using carbon fiber reinforced thermoplastic (CFRP)/aluminum-honeycomb sandwiches with seven layers for the cross panels, which are very stiff, lightweight, and have a very low coefficient of thermal expansion in planar direction. All electronics boxes are mounted on these panels, but outside the instrument compartment, to minimize thermal interaction with the optics. Direct links to the radiators define the main conductive paths and prevent the instrument from disturbances. The request to the thermal system is to hold the optical system in a full-performance range of +15 °C and +25 °C. A rough temperature stabilization is guaranteed by a maintenance heater system. This system consists of thermal switches in combination with heaters to prevent a cooling down of the instrument below around 10 °C. A fine temperature stabilization is achieved by using a dynamic heater system. A group of six heaters in DESIS are controlled by the ICU. The thermal state of the instrument is captured by 16 temperature sensors distributed in the DESIS instrument. With this system, dedicated points of the instrument (radiators, LED banks) can be controlled in an order of 500 mK. A third system exists for the detector. The temperature of the detector is controlled by a Peltier controller. So the temperature can be regulated in both directions with an accuracy of 100 mK. The nominal operation temperature of the detector is 20 °C. With the exception of these different control systems, the thermal control system is mainly a passive system. No multilayer insulation (MLI) is used outside nor inside the container.

## 5. On-Ground Calibration and Testing

To characterize the instrument and to check whether the requirements are fulfilled, an extensive calibration program was executed before launch. The calibration steps were performed at the system level but also at sub-system level. A dedicated description of calibration results can be found in [22,23]. The following items were characterized:

- quantum efficiency (QE), linearity, dark signal non-uniformity (DNSU), photo response non-uniformity (PRNU), dark current noise
- photon transfer curve (PTC)
- modulation transfer function (MTF)
- spectral calibration of the order sorting filter
- wavefront deformation Peak to Valley (PV) and root-mean-square (RMS) of the optical system
- grating efficiency and 2nd order stray-light suppression by grating subsystem
- pointing accuracy and repeatability of pointing unit
- focusing and focus check
- spatial MTF and spectral full width at half maximum (FWHM)
- absolute radiometric calibration for spectral radiances
- spectral response function for each pixel
- keystone and smile
- polarization

These investigations were performed mostly before the final environmental testing of the instrument (thermal vacuum testing, vibration testing). However, some of them were also performed after or during the thermal vacuum tests. Satellite projects also foresee an extensive testing program for the interface between instrument and satellite bus. For the ISS, the situation is different. The flight model of the sensor system has never been tested with the MUSES flight model on ground. Special emulators for the simulation of the interface between MUSES and DESIS were developed. These emulators represent the mechanical, electrical, and functional interface. All instrument tests and all environmental tests were performed with the MUSES emulator.

## 6. Summary and Outlook

**Figure 13:**
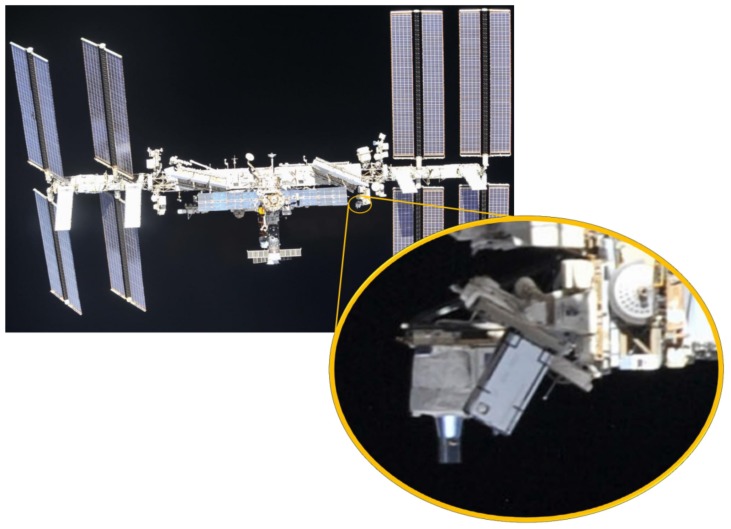
DESIS operating on MUSES onboard of the International Space Station (ISS), photo taken 4 October 2018 from Sojus MS-08. Credit: NASA.

**Figure 14:**
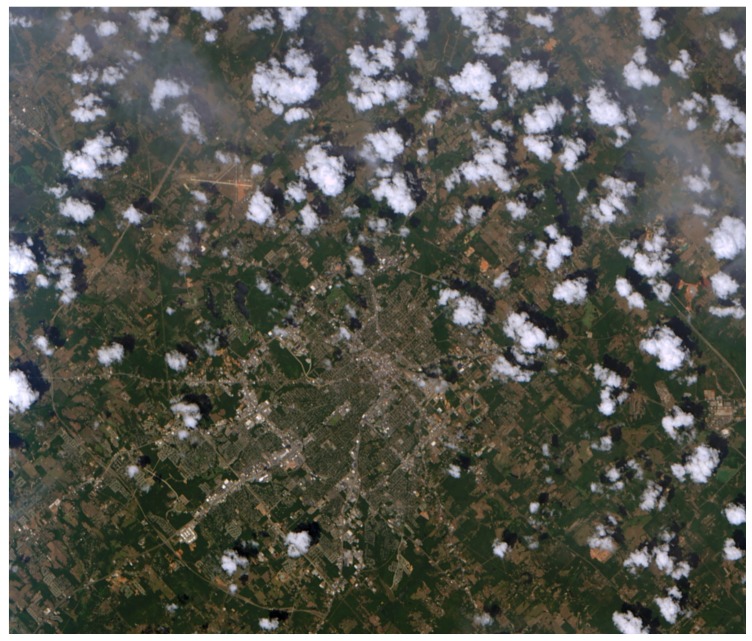

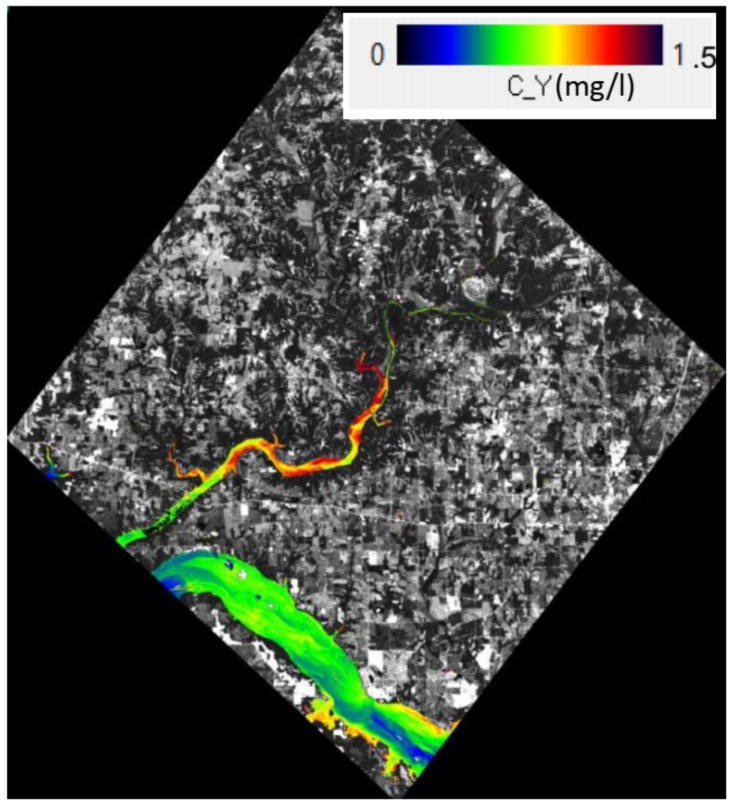
DESIS First Images. (**a**) RGB composition of the first image from Tyler, Texas, U.S., taken on 31 August 2018. (**b**) Colored dissolved organic matter (CDOM) in Huntsville, Alabama, U.S., Tennessee-River.

In this article the design of the DESIS instrument was presented. The DESIS instrument is a hyperspectral instrument onboard the ISS. The instrument has 235 spectral bands in the wavelength range from visible (400 nm) to near-infrared (1000 nm), which results in a 2.5 nm spectral sampling distance and a ground sampling distance of 30 m from 400 km orbit of the ISS. The design was driven by the optical performance, the operating environment onboard the ISS, and the short development time. Right after the installation of the instrument in August 2018, the commissioning phase of the instrument was started (see Figure 13). In this phase, the functionality of the instrument (checkout phase), the optical performance of the instrument, the functionality of the on-ground processors, and the whole infrastructure from the tasking of a target to the delivery of the final product were tested. The first picture of DESIS was taken shortly after installation (see Figure 14a). In Figure 14b, also a first derived product (not groundtruthed yet), the concentration of dissolved organic matter in water is shown [24]. Up to January 2019, as part of the commissioning phase, over 300 data takes with over 950 GB of data were captured. The onground calibration results and the commissioning results will be presented in a separate article.

## Figures and Tables

**Table 1:** German Aerospace Center (DLR) Earth Sensing Imaging Spectrometer (DESIS) parameters.

Parameter	Value
$F_{\#}$	2.8
focal length	320 mm
field of view	4.1°
instantaneous field of view	0.004°
ground sampling distance	30.0 m
spatial pixels	1024
swath	30 km
spectral range	400 nm–1000 nm
spectral channels	235
spectral sampling distance	2.55 nm
spectral binning modes	1, 2, 3, 4
signal-to-noise ratio (albedo 0.3, 45° SZA, 232 Hz @ 550 nm)	195 (no binning) 386 (binning 4)
radiometric linearity	>95% (10–90% full well capacity)
radiometric resolution	12 bit + 1 bit gain
modular transfer function value at Nyquist	>20%
full width at half maximum	<3 nm
pixel size	24 μm × 24 μm
maximum frame rate	232 Hz in Rolling Shutter Mode
polarization	<5%
along track pointing capability	±15°
pointing accuracy	<0.004°
mass	88 kg
ISS orbit	400 km
